# Does an elite education benefit health? Findings from the 1970 British Cohort Study

**DOI:** 10.1093/ije/dyw045

**Published:** 2016-05-10

**Authors:** David Bann, Mark Hamer, Sam Parsons, George B Ploubidis, Alice Sullivan

**Affiliations:** 1 Centre for Longitudinal Studies, UCL Institute of Education, London, UK; 2 School of Sport, Exercise and Health Sciences, Loughborough University, Loughborough, UK; 3 Department of Epidemiology & Public Health, University College London, London, UK

**Keywords:** Socioeconomic factors, education, social determinants of health, cohort studies

## Abstract

**Background:** Attending private school or a higher-status university is thought to benefit future earnings and occupational opportunities. We examined whether these measures were beneficially related to health and selected health-related behaviours in midlife.

**Methods:** Data were from up to 9799 participants from the 1970 British birth Cohort Study. The high school attended (private, grammar or state) was ascertained at 16 years, and the university attended reported at 42 years [categorised as either higher (Russell Group) or normal-status institutions]. Self-reported health, limiting illness and body mass index (BMI) were reported at 42 years, along with television viewing, take-away meal consumption, physical inactivity, smoking and high risk alcohol drinking. Associations were examined using multiple regression models, adjusted for gender and childhood socioeconomic, health and cognitive measures.

**Results:** Private school and higher status university attendance were associated with favourable self-rated health and lower BMI, and beneficially associated with health-related-behaviours. For example, private school attendance was associated with 0.56 [95% confidence interval (CI): 0.48, 0.65] odds of lower self-rated health [odds ratio (OR) for higher-status university: 0.32 (0.27, 0.37)]. Associations were largely attenuated by adjustment for potential confounders, except for those of private schooling and higher-status university attendance with lower BMI and television viewing, and less frequent take-away meal consumption.

**Conclusions:** Private school and higher-status university attendance were related to better self-rated health, lower BMI and multiple favourable health behaviours in midlife. Findings suggest that type or status of education may be an important under-researched construct to consider when documenting and understanding socioeconomic inequalities in health.

## Introduction

Education is thought to benefit future health and economic outcomes,^1–3^ but there is substantial heterogeneity in the latter depending on the type or status of education attended. For example, the minority of persons who attend private high schools in Britain (typically around 7%)^4^ are greatly over-represented in higher-status occupations (e.g. 71% of senior judges, 50% of diplomats),^5^ and typically earn substantially more in adulthood.^6^ Attending a higher rather than normal-status university has also been favourably associated with both occupational opportunities and higher subsequent earnings.^7–9^ Given the importance of occupation and financial resources for health and its behavioural determinants,^10^^,^^11^ private school and higher-status university attendance may also favourably relate to adult health outcomes.

Large inequalities in health are known to exist according to educational attainment,^2^^,^^12^ yet whether attending elite institutions during school and university benefits adult health outcomes is unclear. By having the financial resources to provide improved access to recreational facilities, a higher teacher:pupil ratio, access to beneficial social networks and/or by aiding the development of cognitive and social assets, attending elite institutions may affect adult health through a number of different pathways.^13–16^ In addition, elite institutions may differ in the composition of pupils’ psychosocial characteristics and cultural norms, which could have lasting effects on health-impacting behaviours.^17^ Understanding these relations is important to better understand the socioeconomic distribution of health, and may yield insights into the importance of education quality for population health. A small number of studies in the USA have found that indicators of higher education quality in school are beneficially associated with subsequent adult health.^18^ These suggest that efforts to improve education quality may benefit population health, independently of improvements in the amount of education achieved. For example, improvements in indicators of higher education quality (higher teacher:pupil ratio and teachers’ wages) during the early 20^th^ century have been associated with lower premature mortality risk.^18^^,^^19^ However, these studies lack data for potentially important confounding factors which are likely to select persons into elite institutions (such as early-life socioeconomic position (SEP)^20^ and childhood cognition^21^), and may have conflated the benefits of higher education quality with other societal changes which improved health. A small number of studies, mostly in the economics literature, have examined how school or university characteristics relate to a limited number of adult health-related outcomes.^22–24^ These include the investigation of the type of school and the pupil:teacher ratio,^23^ and selectivity of universities in the USA.^22^^,^^24^ None has examined both school and university type, and important potential confounders such as parental income^23^ have in some cases not been accounted for. Both school type and university status warrant investigation—university, like schooling, is likely to be a period in which patterns of health-impacting behaviours are established, which may subsequently track across life and impact on health.^25–27^

We examined whether private high school or a higher-status university attendance were related to health outcomes, using the 1970 British Cohort Study (BCS70), a large nationally representative sample with detailed data on education, multiple health-related outcomes in early midlife and important potential confounders. We hypothesized that private school and higher-status university attendance would both be beneficially associated with health (and related behaviours), and that associations would be only partly explained by preceding socioeconomic,cognitive and health characteristics.

## Methods

### Study sample

**Figure 1:**
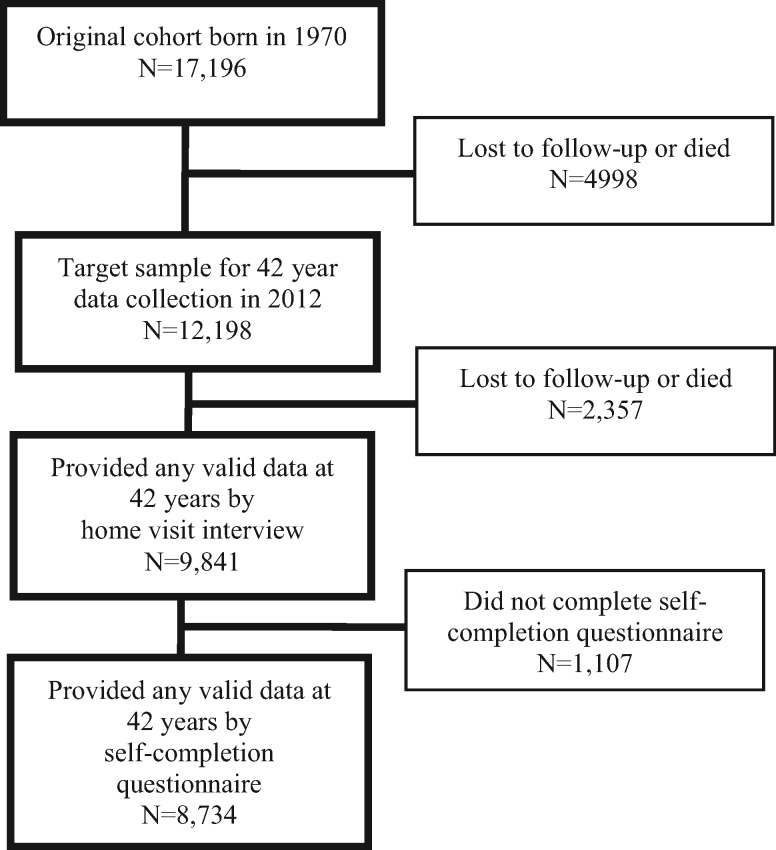
A flow chart summarising response for those who provided valid data at 42 years (2012) in the 1970 British Cohort Study.

The BCS70 consists of all 17 196 babies born in Britain during one week of March 1970, with eight subsequent waves of follow-up from childhood to early midlife.^28^ At the most recent wave (42 years), 12 198 eligible participants (those alive and not yet lost to follow-up) were invited to be interviewed at home by trained research staff—9841 participants (80.7%) responded and provided some valid data, including information on self-rated health, height and weight, and smoking status; 8734 (71.6%) completed an additional self-completion questionnaire, including information on diet and alcohol intake and time spent watching television and undertaking physical activity (see Figure 1 for flow diagram). Previous analyses have found that those of lower SEP in childhood were less likely to provide data at 42 years and at other adult sweeps.^29^ The available analytical sample sizes therefore differed by mode of assessment and by missing data for specific questionnaire items (available sample sizes for all outcomes shown in Tables 1-3). At all waves, informed consent was provided and ethical approval granted.

**Table 1.** Sample characteristics stratified by type of high school and university education

		**High school attended:**			**University attended**		
**Characteristics, and outcomes at 42 years**	***N***	**Comprehensive**	**Grammar**	**Private**	**Did not attend university**	**Normal-status**	**Higher-status**
Total sample, *N* (%)	9658	8651 (89.6%)	389 (4.0%)	618 (6.4%)	7344 (74.6%)	1865 (19.0%)	632 (6.4%)
Women, *N* (%)		4556 (52.7%)	196 (50.4%)	286 (46.3%)**	3809 (51.9%)	991 (53.1%)	317 (50.2%)
Attended any university, *N* (%)		1891 (21.9%)	179 (46.0%)	402 (65.0%)**		–	–
Attended elite university, *N* (%)		396 (4.6%)	46 (11.8%)	183 (29.6%)**		–	–
Parental education: degree (5y), *N* (%)	7658	866 (12.5%)	92 (30.4%)	230 (51.0%)**	554 (9.5%)	414 (28.3%)	236 (45.7%)**
Non-manual parental work (10y), *N* (%)	8161	2917 (39.6%)	213 (65.5%)	399 (85.4%)**	2191 (35.6%)	976 (61.8%)	403 (73.8%)**
High household income (10y), *N* (%)	7747	4507 (64.1%)	223 (74.8%)	379 (89.6%)**	3638 (61.9%)	1080 (73.5%)	448 (86.5%)**
Reading score (10y), mean (SD)	7177	41.13 (11.9)	49.64 (10.4)	50.5 (10.2)**	39.3 (11.9)	48.4 (9.8)	52.3 (8.6)**
Maths score (10y), mean (SD)	7178	44.7 (11.5)	53.4 (10.3)	54.5 (11.2)*	42.9 (11.3)	51.8 (10.0)	56.4 (9.3)**
Reported health difficulty (10y), *N* (%)	7178	2844 (37.7%)	108 (32.5%)	144 (30.3%)**	2430 (38.6%)	554 (34.3%)	170 (30.6%)**
Body mass index (kg/m^2^, 10y), mean (SD)	7580	16.9 (2.1)	16.6 (1.9)	16.9 (2.0)	16.9 (2.1)	16.8 (2.1)	16.8 (1.9)
Fair-poor self-rated health, *N* (%)	9651	1334 (15.4%)	58 (14.9%)	52 (8.4%)**	1281 (17.5%)	159 (8.5%)	48 (7.6%)**
Long-standing illness, *N* (%)	9616	2498 (29.0%)	123 (31.7%)	154 (25.0%)*	2192 (30.1%)	500 (26.9%)	153 (24.3%)**
Body mass index ≥ 25, *N* (%)	8716	4679 (59.8%)	162 (47.0%)	255 (47.0%)**	4067 (61.6%)	847 (50.4%)	253 (44.9%)**
Frequent take-away consumption, *N* (%)	8480	2008 (26.5%)	55 (15.8%)	96 (17.4%)**	1770 (27.8%)	321 (19.3%)	98 (17.0%)**
Watch television > 3 h/day, *N* (%)	8473	2080 (27.5%)	53 (15.2%)	61 (11.1%)**	1987 (31.3%)	224 (13.4%)	46 (7.9%)**
Physically inactive, *N* (%)	8373	2859 (38.1%)	120 (34.8%)	169 (31.7%)**	2550 (40.5%)	504 (30.8%)	147 (26.1%)**
Current smoker, *N* (%)	9652	2275 (26.3%)	85 (21.9%)	112 (18.1%)**	2207 (30.2%)	256 (13.7%)	67 (10.6%)**
Higher-risk alcohol drinking,^a^*N* (%)	8585	1842 (24.0%)	81 (23.1%)	143 (25.7%)	1572 (24.4%)	385 (22.8%)	142 (24.3%)

*N* restricted to those with valid school data; high household income defined as ≥ £100 per week; health difficulty defined as that which resulted in 1 week or more of missed school; y, years.

a Score of 5 or more on the Alcohol Use Disorders Identification Test Primary Care (AUDIT-PC) scale

*P < 0.05, **P < 0.01, calculated using chi-square or t tests across school or university groups.

### Outcome measures

Multiple measures of self-reported physical health and health-related behaviours ascertained at 42 years were included. These included self-rated health (poor, fair, good, very good and excellent), limiting illness or disability (none, classified to a certain extent or severely hampered)^30^and body mass index [derived using self-reported height and weight (kg/m^2^); categorised as  < 25, ≥25-29.9 and ≥30]. Physical inactivity was defined by asking participants to report the frequency of participation in 14 activities during leisure time [classified as ‘inactive’ (0) or active (≥ 1 per week)]. Time typically spent watching television during weekdays, an indicator of sedentary behaviour, was ascertained (none, <1 h, 1-3, 3-5 and >5 h/day), and an indicator of dietary behaviour was ascertained by asking participants the frequency of eating take-away meals (several times a week or more, once or twice a week, at least once a month, less often, or never).^31^ Additional dietary outcomes included the frequency of consumption of home-cooked and convenience meals. Finally, smoking status (current vs non-smoker) and high risk alcohol drinking behaviour were ascertained (defined as a score of 5 or more on the Alcohol Use Disorders Identification Test Primary Care (AUDIT-PC) scale).^32^ Since associations between education and alcohol consumption can differ according to volume consumed and consumption patterns,^33^ additional analyses were performed using total consumption (units in the past 7 days) and consumption frequency when drinking(0 to  ≥ 10 drinks per day).

### Ascertainment of school and university attended

The type of high school attended (from 11–16 years of age) was derived from interviews with school headmasters and from census records at 16 years, or recalled at 42 years if not available, as previously described.^34^ School type was categorized as comprehensive and other types, grammar schools or private schools; those who attended schools for students with special educational needs were not included in analyses (*N* = 109), nor were those with missing data for school type (*N* = 74). Participants’ first university attended was recalled at 42 years and categorized as either higher-status (Russell Group and two other consistently high-ranking institutions) or normal-status universities (all other institutions), as previously described.^34^ The Russell Group is a self-selected group representing 24 purportedly leading universities, and attendance has been related to higher graduate income.^8^

### Analytical strategy

We first tabulated outcome variables by education type, and examined associations with outcomes using chi-square and t tests. Next, we used multiple regression models to examine associations between type of education and health-related outcomes. Binary or ordered logistic models were used, and the proportional odds assumption was tested using a likelihood ratio test to compare constrained and unconstrained models. Models were first adjusted for sex, then additionally for a series of potential confounders chosen a priori (identified from previous analyses of BCS70^34^): indicators of childhood SEP [paternal occupational class and household income (10 years), and education (5 years)], childhood ascertained at 10 years of age (reading and maths scores at 10 years) and childhood health at 10 years of age (school absence due to illness or emotional problems, disability as judged by health visitor). Models examining university education were additionally adjusted for school type. Gender differences in associations were tested by including gender x education interaction terms. For participants with valid outcome data (e.g. self-rated health at 42 years), 424 (4.4%) had missing data for all potential confounders, and 4528 (46.9%) for at least one. To avoid loss of power and to potentially limit bias, we used full-information maximum likelihood estimation (FIML) to account for these missing data and did not further limit analyses to those with valid data for all outcomes (at the expense of comparability across outcomes).

### Additional and sensitivity analyses

We conducted a range of sensitivity analyses to check the robustness of findings. Since education systems differ in Scotland (e.g. few grammar schools exist), we excluded participants from Scotland to examine the extent to which comparisons were conflated with country differences. To examine whether associations with television viewing reflected the entire week’s leisure time, we repeated analyses examining weekend television viewing as an outcome. We also repeated analyses using both stricter definitions of physical inactivity, and more refined (graded) leisure-time physical activity measures. To examine whether additional adjustment for potential confounders affected findings, models were conducted with additional adjustment for: SEP indicators (housing tenure and crowding/persons per room at 5 years of age); other childhood characteristics [measured BMI, social and emotional traits at 10 years of age (self-esteem, locus of control, Rutter behaviour scores, externalizing behaviour, sociability, emotionality, conscientiousness)]; additional cognitive measures at 5 and 10 years of age, as previously described^34^; and maternal characteristics (maternal BMI, teacher’s report of maternal interest in the child’s education, and malaise ascertained at 10 years).

## Results

A total of 618 participants (6.4%) attended private school, and 632 (28.5%) of participants who attended university did so at a higher-status institution (Table 1). Men were more likely to attend private school. Both private school and higher-status university attendance were related to indicators of higher childhood SEP (higher paternal education, occupational class and income), higher reading and maths scores and fewer reported health problems. Grammar school attendance was associated with these measures in the same direction, yet typically more weakly.

### Self-rated health and BMI

Compared with attendance at comprehensive school, private school attendance was associated with better self-rated health [odds ratio (OR) of being in one category worse self-rated health 0.56; 95% confidence interval (CI): 0.48, 0.65], lower odds of limiting illness, and lower BMI (Table 2). When analysed as a continuous outcome, mean differences in BMI were −1.81 (−2.26, −1.36) for private and −1.09 (−1.64, −0.53) for grammar school attendance. After adjustment, associations of private school attendance with self-rated health and limiting illness were largely attenuated, and associations with lower BMI remained (OR 0.71: 0.60, 0.84). Associations with BMI also remained after adjustment for future educational attainment (Supplementary Table 1, available as Supplementary data at *IJE* online).Grammar school attendance was also associated with lower BMI before and after adjustment (Table 2).

**Table 2.** High school attended in relation to self-reported health and health-related behaviours at 42 years

		**High school attended:**		
**Outcomes at 42 years**	*N*	Comprehensive Ref.	Grammar OR (95% CI)	Private OR (95% CI)
Sex-adjusted models				
Lower self-rated health	9651	–	0.89 (0.74, 1.07)	0.56 (0.48, 0.65)
Long-standing illness	9616	–	0.87 (0.65, 1.18)	0.60 (0.45, 0.79)
Higher body mass index (kg/m^2^)	8716	–	0.61 (0.49, 0.75)	0.53 (0.45, 0.62)
Frequent take-away consumption	8480	–	0.59 (0.49, 0.71)	0.54 (0.46, 0.63)
Higher television viewing	8473	–	0.54 (0.44, 0.66)	0.38 (0.32, 0.45)
Physically inactive	8373	–	0.87 (0.69, 1.09)	0.75 (0.62, 0.90)
Current smoker	9652	–	0.78 (0.61, 1.00)	0.62 (0.50, 0.76)
Higher-risk alcohol drinking	8585	–	0.93 (0.72, 1.20)	1.03 (0.84, 1.26)
Fully adjusted models^a^				
Lower self-rated health	9651	–	1.24 (1.02, 1.50)	0.92 (0.78, 1.09)
Long-standing illness	9616	–	1.07 (0.79, 1.46)	0.87 (0.64, 1.17)
Higher body mass index (kg/m^2^)	8716	–	0.73 (0.59, 0.91)	0.71 (0.60, 0.84)
Frequent take-away consumption	8480	–	0.69 (0.57, 0.84)	0.68 (0.58, 0.81)
Higher television viewing	8473	–	0.83 (0.68, 1.02)	0.67 (0.56, 0.80)
Physically inactive	8373	–	1.05 (0.83, 1.33)	1.00 (0.82, 1.22)
Current smoker	9652	–	1.07 (0.83, 1.38)	1.00 (0.79, 1.25)
Higher-risk alcohol drinking	8585	–	0.86 (0.66, 1.12)	0.89 (0.72, 1.11)

a Adjusted for childhood socioeconomic indicators [paternal occupational class and household income (10 years), and education (5 years)], childhood cognition (reading and maths scores at 10 years) and childhood health (school absence due to illness or emotional problems, disability as judged by health visitor at 10 years).

Higher-status university attendance was associated with better self-rated health and lower BMI, but not with limiting illness (Table 3)—compared with normal-status university attendance, the odds of being in a higher (heavier) BMI category were 0.75 (0.63, 0.89)among those who attended a higher-status university (mean difference in BMI = −0.74; −1.22, −0.25). After adjustment, differences in self-rated health were largely attenuated, whereas those with BMI largely remained.

**Table 3.** University attended in relation to self-reported health and health-related behaviours at 42 years

		**University attended:**			
**Outcomes at 42 years**	***N***	**None, no qualifications OR (95% CI)**	**None, pre-university qualification OR (95% CI)**	**Normal-status university Ref**	**Higher-status university OR (95% CI)**
Sex-adjusted models					
Lower self-rated health	9799	2.55 (2.29, 2.84)	1.70 (1.54, 1.87)	–	0.76 (0.64, 0.90)
Long-standing illness	9763	2.25 (1.90, 2.67)	1.37 (1.16, 1.62)	–	0.86 (0.64, 1.17)
Higher body mass index (kg/m^2^)	8841	1.71 (1.53, 1.92)	1.57 (1.42, 1.75)	–	0.75 (0.63, 0.89)
Frequent take-away consumption	8617	1.64 (1.46, 1.85)	1.50 (1.35, 1.67)	–	0.73 (0.61, 0.87)
Higher television viewing	8604	2.95 (2.61, 3.34)	2.11 (1.90, 2.34)	–	0.60 (0.51, 0.71)
Physically inactive	8503	1.75 (1.53, 2.00)	1.41 (1.25, 1.60)	–	0.79 (0.64, 0.99)
Current smoker	9801	3.69 (3.17, 4.30)	2.18 (1.88, 2.53)	–	0.74 (0.56, 0.99)
Higher-risk alcohol drinking	8721	1.12 (0.97, 1.30)	1.05 (0.92, 1.21)	–	1.04 (0.83, 1.31)
Fully adjusted models					
Lower self-rated health	9799	1.91 (1.69, 2.14)	1.45 (1.31, 1.61)	–	0.91 (0.76, 1.08)
Long-standing illness	9763	1.86 (1.55, 2.25)	1.25 (1.05, 1.49)	–	1.00 (0.73, 1.36)
Higher body mass index (kg/m^2^)	8841	1.36 (1.19, 1.54)	1.36 (1.22, 1.53)	–	0.85 (0.72, 1.02)
Frequent take-away consumption	8617	1.37 (1.20, 1.56)	1.33 (1.19, 1.49)	–	0.81 (0.68, 0.98)
Higher television viewing	8604	1.89 (1.66, 2.16)	1.63 (1.46, 1.82)	–	0.78 (0.65, 0.93)
Physically inactive	8503	1.53 (1.32, 1.77)	1.32 (1.16, 1.51)	–	0.85 (0.68, 1.07)
Current smoker	9801	2.95 (2.50, 3.48)	1.93 (1.66, 2.25)	–	0.82 (0.61, 1.10)
Higher-risk alcohol drinking	8721	1.27 (1.08, 1.50)	1.12 (0.96, 1.29)	–	0.97 (0.77, 1.22)

Fully adjusted models adjusted for childhood socioeconomic indicators [paternal occupational class and household income (10 years), and education (5 years)], childhood cognition (reading and maths scores at 10 years) and childhood health (school absence due to illness or emotional problems, disability as judged by health visitor at 10 years) and school type.

### Selected health-related behaviours

Private school attendance was associated with greater odds of being physically active, less frequent television viewing, lower odds of smoking, and less frequent take-away meal consumption. After full adjustment, these associations were largely attenuated, except for those with lower television viewing and take-away meal consumption (Table 2). Associations were only partly attenuated after adjustment for future educational attainment (Supplementary Table 1, available as Supplementary data at *IJE* online). School attendance was not associated with high-risk alcohol drinking, but private school attendance was associated with higher consumption of alcohol units, yet with lower number of drinks consumed on a given drinking day; only the latter association remained after confounder adjustment (Supplementary Table 2, available as Supplementary data at *IJE* online). Associations between grammar school attendance and outcomes were typically similar to those of private school.

Higher-status university attendance was also associated with greater odds of physical activity, less frequent television viewing, lower odds of smoking and with less frequent take-away meal consumption, yet not with high-risk alcohol drinking (Table 3). After adjustment, higher-status university attendance was associated with lower television viewing and less frequent take-away consumption, whereas associations with other outcomes were attenuated. Higher-status university attendance was also associated, after adjustment, with lower alcohol consumption on a given day of drinking (Supplementary Table 3, available as Supplementary data at *IJE* online).

For all outcomes, we found little evidence for gender interaction (*P* > 0.11 for interaction terms). Findings were similar when excluding participants residing in Scotland, when using television viewing during weekends as an outcome, when using alternative physical activity measures and when adjusting for additional potential confounders (Supplementary Tables 2_5, available as Supplementary data at *IJE* online).

## Discussion

In a large nationally representative birth cohort study initiated in 1970, private school and higher-status university attendance were beneficially and independently related to self-rated health, lower BMI and selected health-related behaviours (physical inactivity, smoking, television viewing and take-away meal consumption). Associations with BMI, television viewing and take-away meal consumption were robust to adjustment for potential confounders, whereas associations with other outcomes were largely explained by these factors.

The current findings suggest that the type or status of education may be an important part of the socioeconomic environment which has thus far not been well researched in studies of health inequality. They add to studies demonstrating inequalities in health outcomes according to educational attainment^1^^,^^35^^,^^36^ and to studies examining how aspects of schooling or university education are related to a more limited number of health outcomes.^22–24^ By not accounting for the type or status of education, and focusing solely on attainment, it is possible that existing epidemiological studies have underestimated differences in health attributable to education.

The persistence of beneficial associations with BMI, television viewing, and take-away meal consumption may be suggestive of causal relationships between the type of education and these adiposity-related outcomes. Although we adjusted for a large number of prospectively ascertained potential confounders, residual confounding cannot be entirely ruled out given the strong links between potential confounders and these outcomes (e.g. childhood SEP and both adiposity^37^^,^^38^ and television viewing^39^), nor the potential of confounding due to unobserved family characteristics (e.g. parental ambition). Different study designs could be used to aid causal inference in future studies. For example, situations in which private school places are selected randomly, and therefore independent of potential confounders such as parental socioeconomic circumstances, could be utilised. Existing studies of this nature have not typically found strong effects of voucher allocation on short-term education outcomes (e.g. in India and the USA).^40^^,^^41^ However, the short-term follow-up of these studies and country differences in school characteristics limits generalizability to Britain, where private education is thought to yield substantial long-term economic returns in adulthood.^6^^,^^42^

Although the causal nature of the relations found in this study are uncertain, there are a number of pathways which could feasibly underlie causal relations between type of education and health—including socioeconomic and behavioural pathways—and these warrant future investigation. Private schools are likely to be more financially resourced to provide a greater quantity and quality of extracurricular activities. For example, in BCS70, the mean number of different extracurricular activities typically available to students was 16 in comprehensive, 15 in grammar and 25 in private schools (*P* < 0.001, data available on request). These differences may have been specially large during the 1980s, when teachers’ industrial action in the state sector included strikes and a ‘work to rule’ which could have limited extracurricular activities. The composition of both teachers and pupils differs between private and state schools, and both may have also contributed to peer effects on pupils’ behavioural patterns.^16^^,^^43^^,^^44^ Due to tracking, such behaviours may persist into adult life and ultimately affect BMI and other health outcomes.^25^^,^^45^ Although private school attendees were more likely to have gone on to attain higher subsequent education (Table 1),^34^ educational attainment did not fully explain the observed associations. However, other aspects of adult socioeconomic circumstances may have a role in explaining these associations. For example, accrued benefits in earnings may have contributed to these differences, given its expected benefits on diet quality and aspects of cultural consumption which displace television viewing. Associations with private schooling could also partly reflect the influence of area-level socioeconomic circumstances known to be related to adult BMI, diet and activity participation^31^^,^^46^^,^^47^; grammar and private school attendees have also been found to be more likely to move to areas with favourable measures of population health.^48^

For other outcomes, beneficial associations of school and university type with outcomes were largely explained by adjustment for potential confounders. Childhood socioeconomic and cognitive measures appeared to play particularly important roles in attenuating the observed relationships, whereas childhood health or BMI measures did not. Indeed, childhood BMI did not differ between school or university groups. This suggests that the type of education acted as a powerful marker of preceding socioeconomic and cognitive characteristics which in turn related to health outcomes and behaviours.^20^^,^^21^ However, relations between type of education and outcomes may differ by age. For example, genuine differences in BMI and television viewing may eventually result in clinically manifest differences in health and disability risk in older age.^49^^,^^50^ Associations between education type and alcohol consumption differed by the alcohol measure used—for example, elite university attendance was associated with lower odds of problematic alcohol consumption, yet with higher total intake. These divergent relationships are consistent with studies examining educational attainment in relation to alcohol,^33^ and the net health effects warrant future investigation.

Compared with attending private school, grammar school attendance was less strongly associated with favourable self-reported health and behavioural outcomes in unadjusted models. Grammar schools are free alternatives to state funded schools which purportedly selected students on the basis of higher student performance. Most, but not all, were converted to state or private schools in the 1970-80s following educational reforms.^51^ Stronger associations between private schooling and outcomes in unadjusted models may therefore be driven by stronger selection into private schools among participants with more advantaged socioeconomic backgrounds and with higher cognitive scores (Table 1). Once these factors were adjusted for, effect estimates for grammar and private school were similar, and confidence intervals overlapped. This suggests a similar potential benefit of private and grammar schooling for lower BMI and less frequent take-away food consumption. In BCS70, grammar school attendance was not found to be associated with the likelihood of obtaining a university degree (in adjusted models),^34^ suggesting that pathways relating to these health outcomes are unlikely to be explained by educational attainment. However, the number of participants in this group was especially small (4%), which may limit the power to detect genuine differences in outcomes.

Strengths of this study include the large nationally representative sample, with rich data for school and university type, multiple health-related outcomes and detailed data for childhood characteristics which were found to be important in explaining some of the observed relationships. Although multiple outcomes were used, the measures of health and behaviour were not comprehensive—further research is required to examine relations between type of education and the multiple dimensions of physical activity and dietary behaviour, which may have distinct importance for health outcomes. Although many of the self-reported measures used have been shown to predict important objective outcomes,^52^ their use may have contributed to bias if the propensity to misreport differed by educational group. The direction and magnitude of bias is likely to depend on the outcome used. For example, higher education attainment has been associated with modest underestimation of BMI, potentially resulting in overestimates of inequalities in BMI,^53^ and yet with overestimation of self-rated health problems, potentially resulting in underestimates of inequalities in health.^54^^,^^55^ Future studies are therefore required to examine relations with objective measures of health and behaviours. Differences may also exist in other aspects of health not investigated in this study, such as mental health and positive mental well-being. As in all longitudinal studies, attrition occurred in the BCS70^29^ although we attempted to account for this by using FIML. Finally, although the school and university groupings were found to relate to the outcomes considered, future studies may be able to examine more refined categorization of school and university type and/or examine other aspects of experience in education.

Notwithstanding the study limitations, there are a number of potential implications. Findings suggest that type of education may be an important construct to consider when documenting and understanding the socioeconomic distribution of health. When examining education attainment either as a main exposure or as a potential confounder, considering type or status of education may yield additional information with relevance for subsequent health outcomes. Findings may also suggest that targeting aspects of schooling which differ between state and private schools may be helpful in reducing BMI and sedentary behaviour in the population and the socioeconomic inequalities in these outcomes which have thus far proved challenging to modify. Although the proportion of privately educated persons has been relatively consistent in Britain (∼7%), the extent to which these findings are generalizable to younger or older birth cohorts requires investigation,^6^ as does generalizability to other countries with different educational systems.

In conclusion, our findings suggest beneficial relations between elite education during schooling and university, with subsequent self-rated health and health-related behaviours in midlife. Although the causal nature of these relations remains to be determined, these findings support the hypothesis that the type or status of education may be an important construct to consider when investigating socioeconomic inequalities in health.

## Supplementary Data

Supplementary data are available at *IJE* online.

## Funding

This work was supported by the Economic and Social Research Council (grant number ES/M008584/1).

Key Messages

- Attending private school or a higher- status university typically benefits future economic outcomes, but it is unclear if these indicators of socioeconomic advantage relate to adult health and behavioural outcomes.
- Using a British cohort study initiated in 1970, private school and higher- status university attendance were beneficially related to self-rated health, lower BMI and multiple health-related behaviours
- Associations with lower BMI and less frequent television viewing and take-away meal consumption were independent of potential confounding factors (childhood cognitive, socioeconomic and health measures).
- In addition to educational attainment, findings suggest that type or status of education may be an important under-researched construct to consider when documenting and explaining socioeconomic inequalities in health.

## Supplementary Material

Supplementary Data
