# Supplementary material for: Does an elite education benefit health? Findings from the 1970 British Cohort Study
Source: Int J Epidemiol. 2016 May 10;46(1):293–302. doi: 10.1093/ije/dyw045 (PMC5407151; doi:10.1093/ije/dyw045)
Supplement: Supplementary Data [file dyw045_supp.docx]

Supplementary Table 1. High school attended in relation to self-reported health and selected health-impacting behaviours at 42 years, additionally adjusted for educational attainment

|  | **High school attended:** |  |  |  |
| --- | --- | --- | --- | --- |
|  | Comprehensive  Ref. | Grammar  OR (95% CI) | Private  OR (95% CI) |  |
| **Outcomes at 42 years** |  |  |  |  |
| Fully adjusted models* |  |  |  |  |
| Lower self-rated health | - | 1.30 (1.07, 1.57) | 1.02 (0.87, 1.20) |  |
| Long-standing illness | - | 1.11 (0.81, 1.53) | 0.93 (0.69, 1.27) |  |
| Higher body mass index (kg/m^2^) | - | 0.77 (0.62, 0.95) | 0.78 (0.65, 0.92) |  |
| Frequent takeaway consumption | - | 0.72 (0.59, 0.87) | 0.75 (0.63, 0.90) |  |
| Higher television viewing | - | 0.88 (0.72, 1.09) | 0.77 (0.65, 0.93) |  |
| Physically inactive | - | 1.10 (0.87, 1.39) | 1.11 (0.90, 1.36) |  |
| Current smoker | - | 1.18 (0.91, 1.52) | 1.21 (0.95, 1.53) |  |
| High risk alcohol drinking | - | 0.88 (0.67, 1.14) | 0.92 (0.74, 1.15) |  |

*adjusted for indicators of childhood socioeconomic indicators (paternal occupational class and household income (10 years), and education (5 years)), childhood cognition (reading and maths scores at 10 years), and childhood health (school absence due to illness or emotional problems, disability as judged by health visitor at 10 years). and adult educational attainment (none, no qualifications, pre-university qualification, normal status university, or higher status university degree).

Supplementary Table 2. High school attended in relation to selected health-related behaviours at 42 years

|  | **High school attended:** |  |  |  |
| --- | --- | --- | --- | --- |
|  | Comprehensive  Ref. | Grammar  OR (95% CI) | Private  OR (95% CI) |  |
| **Outcomes at 42 years** |  |  |  |  |
| Sex-adjusted models |  |  |  |  |
| Lower home cooked meal consumption | - | 0.73 (0.60, 0.89) | 0.57 (0.49, 0.66) |  |
| Lower ready-meal consumption | - | 1.14 (0.96, 1.36) | 1.07 (0.92, 1.24) |  |
| Lower convenience food consumption# | - | 1.16 (0.97, 1.40) | 1.61 (1.38, 1.88) |  |
| Higher alcohol units consumption | - | 1.33 (1.11, 1.59) | 1.51 (1.31, 1.73) |  |
| Higher daily drinks consumed | - | 0.85 (0.72, 1.00) | 0.74 (0.65, 0.84) |  |
| Physical activity i | - | 1.29 (1.04, 1.60) | 1.29 (1.08, 1.54) |  |
| Physical activity ii | - | 1.16 (0.96, 1.41) | 1.45 (1.22, 1.71) |  |
|  |  |  |  |  |
| Fully adjusted models* |  |  |  |  |
| Lower home cooked meal consumption | - | 0.86 (0.70, 1.04) | 0.75 (0.63, 0.89) |  |
| Lower ready-meal consumption | - | 1.11 (0.93, 1.33) | 1.01 (0.86, 1.19) |  |
| Lower convenience food consumption# | - | 1.07 (0.88, 1.29) | 1.34 (1.14, 1.58) |  |
| Higher alcohol units consumption | - | 1.01 (0.84, 1.22) | 1.00 (0.86, 1.17) |  |
| Higher daily drinks consumed | - | 0.86 (0.73, 1.02) | 0.79 (0.68, 0.90) |  |
| Physical activity i | - | 1.14 (0.91, 1.43) | 1.06 (0.88, 1.28) |  |
| Physical activity ii | - | 0.97 (0.80, 1.19) | 1.09 (0.91, 1.30) |  |

*indicators of childhood socioeconomic indicators (paternal occupational class and household income (10 years), and education (5 years)), childhood cognition (reading and maths scores at 10 years), and childhood health (school absence due to illness or emotional problems, disability as judged by health visitor at 10 years). Food consumption variables all categorised as >1 per day, 1 per day, several times a week, 1/2 per week, 1 per month, less often, never. Alcohol units consumed per last 7 days categorised as 0-4, >4-8.7, >8.7-19.7, >19.7 units; daily drinks consumed on a given day of drinking categorised as 0, 1-2, 3-4, 5-6, 7-8, >8. #participants were asked to recall consumption of “other convenience foods, frozen or packaged, such as fish-fingers, burgers, chips or ready-made pizzas”; physically active defined as: i) whether participant participated in leisure activities for 2-3 occasions or more per week; ii) the number of leisure activities undertaken at least once per week (0, 1, 2, 3, ≥4).

Supplementary Table 3. University attended in relation to self-reported health and selected health-impacting behaviours at 42 years

|  | **University attended:** |  |  |  |  |
| --- | --- | --- | --- | --- | --- |
|  | None, no qualifications  OR (95% CI) | None,  pre-university qualification  OR (95% CI) | Normal status university  Ref. | Higher status university  OR (95% CI) |  |
| **Outcomes at 42 years** |  |  |  |  |  |
| Sex-adjusted models |  |  |  |  |  |
| Lower home cooked meal consumption | 1.78 (1.59, 2.01) | 1.46 (1.31, 1.62) | - | 0.86 (0.73, 1.02) |  |
| Lower ready-meal consumption | 0.91 (0.81, 1.01) | 0.95 (0.86, 1.05) | - | 1.20 (1.02, 1.41) |  |
| Lower convenience food consumption# | 0.69 (0.61, 0.77) | 0.75 (0.68, 0.83) | - | 1.19 (1.01, 1.40) |  |
| Higher alcohol units consumption | 0.65 (0.58, 0.72) | 0.85 (0.78, 0.93) | - | 1.17 (1.01, 1.36) |  |
| Higher daily drinks consumed | 1.31 (1.16, 1.49) | 1.19 (1.08, 1.32) | - | 0.76 (0.66, 0.88) |  |
| Physical activity i | 0.72 (0.62, 0.82) | 0.79 (0.70, 0.89) | - | 0.90 (0.74, 1.09) |  |
| Physical activity ii | 0.61 (0.54, 0.70) | 0.71 (0.64, 0.80) | - | 1.12 (0.93, 1.34) |  |
|  |  |  |  |  |  |
| Fully adjusted models* |  |  |  |  |  |
| Lower home cooked meal consumption | 1.53 (1.34, 1.74) | 1.31 (1.18, 1.47) | - | 0.98 (0.82, 1.17) |  |
| Lower ready-meal consumption | 0.92 (0.81, 1.04) | 0.97 (0.87, 1.07) | - | 1.21 (1.02, 1.43) |  |
| Lower convenience food consumption# | 0.74 (0.65, 0.84) | 0.79 (0.71, 0.88) | - | 1.10 (0.93, 1.30) |  |
| Higher alcohol units consumption | 0.91 (0.80, 1.02) | 1.01 (0.92, 1.12) | - | 0.95 (0.82, 1.11) |  |
| Higher daily drinks consumed | 1.31 (1.16, 1.49) | 1.19 (1.08, 1.32) | - | 0.76 (0.66, 0.88) |  |
| Physical activity i | 0.72 (0.62, 0.82) | 0.79 (0.70, 0.89) | - | 0.90 (0.74, 1.09) |  |
| Physical activity ii | 0.61 (0.54, 0.70) | 0.71 (0.64, 0.80) | - | 1.12 (0.93, 1.34) |  |

**adjusted for indicators of childhood socioeconomic indicators (paternal occupational class and household income (10 years), and education (5 years)), childhood cognition (reading and maths scores at 10 years), and childhood health (school absence due to illness or emotional problems, disability as judged by health visitor at 10 years), and school type. Food consumption variables all categorised as >1 per day, 1 per day, several times a week, 1/2 per week, 1 per month, less often, never. Alcohol units consumed per last 7 days categorised as 0-4, >4-8.7, >8.7-19.7, >19.7 units; daily drinks consumed on a given day of drinking categorised as 0, 1-2, 3-4, 5-6, 7-8, >8. #participants were asked to recall consumption of “other convenience foods, frozen or packaged, such as fish-fingers, burgers, chips or ready-made pizzas”; physically active defined as: i) whether participant participated in leisure activities for 2-3 occasions or more per week; ii) the number of leisure activities undertaken at least once per week (0, 1, 2, 3, ≥4).

Supplementary Table 4. High school attended in relation to self-reported health and health-related behaviours at 42 years

|  |  | **High school attended:** | |  | |  | |  |
| --- | --- | --- | --- | --- | --- | --- | --- | --- |
|  | Comprehensive  Ref. | | Grammar  OR (95% CI) | | Private  OR (95% CI) | |  |  |
| **Outcomes at 42 years** |  | |  | |  | |  |  |
| Fully adjusted models* |  | |  | |  | |  |  |
| Lower self-rated health | - | | 1.28 (1.05, 1.55) | | 0.92 (0.78, 1.09) | |  |  |
| Long-standing illness | - | | 1.11 (0.81, 1.51) | | 0.85 (0.63, 1.14) | |  |  |
| Higher body mass index (kg/m^2^) | - | | 0.81 (0.64, 1.02) | | 0.68 (0.57, 0.82) | |  |  |
| Frequent takeaway consumption | - | | 0.72 (0.59, 0.87) | | 0.71 (0.59, 0.84) | |  |  |
| Higher television viewing | - | | 0.88 (0.71, 1.08) | | 0.67 (0.56, 0.81) | |  |  |
| Physically inactive | - | | 1.06 (0.84, 1.35) | | 0.99 (0.81, 1.22) | |  |  |
| Current smoker | - | | 1.12 (0.87, 1.45) | | 0.99 (0.78, 1.24) | |  |  |
| Higher risk alcohol drinking | - | | 0.88 (0.67, 1.14) | | 0.92 (0.74, 1.15) | |  |  |

*adjusted for adjusted for indicators of childhood socioeconomic indicators (paternal occupational class and household income (10 years), and education (5 years)), childhood cognition (reading and maths scores at 10 years), and childhood health (school absence due to illness or emotional problems, disability as judged by health visitor at 10 years); also adjusted for additional SEP indicators (housing tenure and number of persons per room at 5 years); other childhood characteristics (measured BMI, social and emotional traits at 10 years (self-esteem, locus of control, Rutter behaviour scores, externalising behaviour, sociability, emotionality, conscientiousness)); additional cognitive measures at 5 and 10 years, as previously described^32^; and maternal characteristics (maternal BMI, teacher’s report of maternal interest in the child’s education, and malaise at 10 years).

Supplementary Table 5. University attended in relation to self-reported health and health-related behaviours at 42 years

|  |  | **University attended:** | |  | |  | |  | |  |
| --- | --- | --- | --- | --- | --- | --- | --- | --- | --- | --- |
|  | None, no qualifications  OR (95% CI) | | None,  pre-university qualification  OR (95% CI) | | Normal status university  Ref | | Higher status university  OR (95% CI) | |  |  |
| **Outcomes at 42 years** |  | |  | |  | |  | |  |  |
|  |  | |  | |  | |  | |  |  |
| Fully adjusted models* |  | |  | |  | |  | |  |  |
| Lower self-rated health | 1.77 (1.57, 2.00) | | 1.40 (1.26, 1.55) | | - | | 0.91 (0.77, 1.09) | |  |  |
| Long-standing illness | 1.68 (1.39, 2.03) | | 1.19 (1.00, 1.43) | | - | | 1.01 (0.74, 1.38) | |  |  |
| Higher body mass index (kg/m^2^) | 1.32 (1.15, 1.51) | | 1.33 (1.18, 1.49) | | - | | 0.85 (0.70, 1.03) | |  |  |
| Frequent takeaway consumption | 1.36 (1.19, 1.55) | | 1.32 (1.18, 1.47) | | - | | 0.83 (0.69, 1.00) | |  |  |
| Higher television viewing | 1.80 (1.57, 2.05) | | 1.56 (1.39, 1.74) | | - | | 0.78 (0.66, 0.93) | |  |  |
| Physically inactive | 1.44 (1.24, 1.67) | | 1.28 (1.13, 1.46) | | - | | 0.85 (0.68, 1.07) | |  |  |
| Current smoker | 2.73 (2.31, 3.22) | | 1.87 (1.60, 2.19) | | - | | 0.85 (0.63, 1.13) | |  |  |
| Higher risk alcohol drinking | 1.30 (1.10, 1.53) | | 1.13 (0.98, 1.30) | | - | | 1.00 (0.80, 1.26) | |  |  |

*adjusted for adjusted for indicators of childhood socioeconomic indicators (paternal occupational class and household income (10 years), and education (5 years)), childhood cognition (reading and maths scores at 10 years), and childhood health (school absence due to illness or emotional problems, disability as judged by health visitor at 10 years), and school type; also adjusted for additional SEP indicators (housing tenure and number of persons per room at 5 years); other childhood characteristics (measured BMI, social and emotional traits at 10 years (self-esteem, locus of control, Rutter behaviour scores, externalising behaviour, sociability, emotionality, conscientiousness)); additional cognitive measures at 5 and 10 years, as previously described^32^; and maternal characteristics (maternal BMI, teacher’s report of maternal interest in the child’s education, and malaise at 10 years).
